# CRISPR base editing-mediated correction of a tau mutation rescues cognitive decline in a mouse model of tauopathy

**DOI:** 10.1186/s40035-024-00415-y

**Published:** 2024-04-12

**Authors:** Min Sung Gee, Eunji Kwon, Myeong-Hoon Song, Seung Ho Jeon, Namkwon Kim, Jong Kil Lee, Taeyoung Koo

**Affiliations:** 1 College of Pharmacy, Kyung Hee University, Seoul, 02447 Republic of Korea; 2 Department of Biomedical and Pharmaceutical Sciences, Graudate School, Kyung Hee University, Seoul, 02447 Republic of Korea; 3 Department of Pharmaceutical Sciences, College of Pharmacy, Kyung Hee University, Seoul, 02447 Republic of Korea

## Main text

The microtubule-binding protein tau is encoded by *MAPT*, located on chromosome 17. Mutations in this gene have been implicated in frontotemporal dementia [1]. Down-regulation of endogenous tau with antisense oligonucleotides (ASOs) specific for human tau or zinc-finger protein transcription factors has been explored in preclinical models of tauopathy [2, 3]. Of particular note, the effects of tau ASOs on mild Alzheimer’s disease are now under assessment in a clinical trial [4]. In addition, CRISPR-mediated gene knockout has been used to regulate the expression of *APP* or *BACE1* to ameliorate amyloid β and tau pathologies [5, 6]. However, therapeutic approaches to correcting *MAPT* mutations that cause tau aggregation in animal models of tauopathy have not yet been studied.

CRISPR RNA-guided base editors have been recently used for targeted base mutagenesis in the genome and have become a promising approach for the treatment of neurological disorders [6]. The recently developed adenine base editor, NG-ABE8e, which is a fusion of SpCas9-NG derived from *Streptococcus pyogenes* and an evolved *E. coli* TadA monomer that is used in combination with a single-guide RNA (sgRNA), generates A-to-G conversions in the spacer upstream of an NG protospacer adjacent motif (PAM). NG-ABE8e has demonstrated an efficient genome editing ability, targeting a window spanning positions 4–11 in the protospacer [7].

**Fig. 1:**
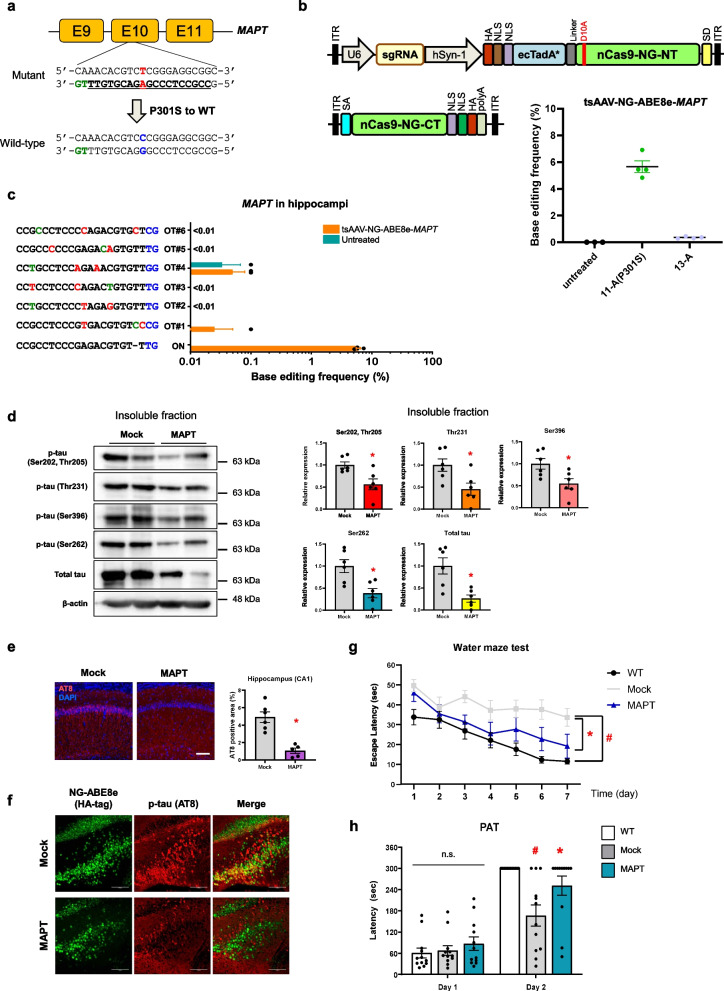
**a** sgRNAs were designed to target exon 10 of the *MAPT*. Protospacer sequences are underlined, PAM sequences are shown in green, the targeted nucleotide in red, and base edited nucleotides in blue. **b** Adenine base editing frequencies induced by tsAAV-NG-ABE8e-*MAPT,* which is controlled by the hSyn-1 promoter, in the hippocampus of PS19 mice at eight weeks after intracranial injection. Error bars indicate SEM (*n* = 4). **c** Genomic DNA isolated from the hippocampi of PS19 mice at eight weeks after injection of tsAAV-NG-ABE8e-*MAPT* was subjected to targeted deep sequencing. Mismatched nucleotides are shown in red, PAM sequences in blue, and DNA bulge on green. ON, on-target site; OT, off-target site. Error bars indicate SEM (*n* = 4). **d** Representative western blots using anti-tau antibodies and quantification of immunoblot staining. All antibodies used in this study are described in Table S4. *P < 0.05 vs mock control, Student’s *t*-test. Error bars indicate SEM (*n* = 6). **e** Representative images and quantification of anti-phospho-tau (AT8) staining in the hippocampus. *P < 0.05 vs mock control, Student’s *t*-test. Scale bar, 100 µm; *n* = 5–6. **f** Representative images of double-staining with anti-phospho-tau (AT8) and anti-HA-tag (which recognizes the NG-ABE8e fusion protein) antibodies in the hippocampus. Scale bars, 100 µm. **g** The escape latency during the training phase in the water maze test. #*P* < 0.05 vs WT, *P < 0.05 vs mock control; generalized estimating equation analysis. WT, *n* = 12; Mock, *n* = 11; MAPT, *n* = 10. **h** The latency to enter the dark compartment in the passive avoidance test (PAT). Maximum time was 300 s. #*P* < 0.05 vs WT, *P < 0.05 vs mock control, One-way ANOVA. WT, *n* = 12; Mock, *n* = 12; MAPT, *n* = 12

In this study, we examined whether NG-ABE8e could be used to correct a pathogenic *MAPT* mutation and thereby reduce tauopathy and cognitive symptoms in the PS19 transgenic mouse model expressing human *MAPT-*P301S. To evaluate the ability of NG-ABE8e to correct the *MAPT*-P301S mutant allele to the wild-type (WT) sequence, we designed sgRNAs targeting the *MAPT*-P301S mutation. The sgRNAs were designed to hybridize with a 19-nt target sequence upstream of a TG PAM to replace the A, located 11 nt distal from the 5′-end of protospacer (Fig. 1a and Additional file 1: Table S1). Next, we evaluated the activity of the sgRNA by using targeted deep sequencing to measure adenine base editing frequencies after transfection of plasmids encoding NG-ABE8e and the sgRNAs into HEK293T cells harboring the P301S mutation (293T-P301S) (Additional file 1: Fig. S1a). The desired A-to-G substitution induced by NG-ABE8e corrected the mutant allele to the WT *MAPT* sequence, with an observed editing frequency of 16.6% ± 0.8% in the cells (Additional file 1: Fig. S1b). Bystander editing or indels were not detectable in the protospacer. We also designed sgRNAs to target exon 1 in the mouse *Rosa26* gene as an internal control (Additional file 1: Fig. S1c and Table S1). Treatment of NIH3T3 cells with NG-ABE8e and a *Rosa26*-targeting sgRNA resulted in a base-editing frequency of 29.4% ± 1.3% (Additional file 1: Fig. S1d).

To evaluate the ability of NG-ABE8e to reduce tau aggregation in vivo, we delivered NG-ABE8e to the hippocampi of PS19 mice. As there was no prior reports on the in vivo delivery of NG-ABE8e using an adeno-associated virus (AAV) vector, we employed an RNA trans-splicing (ts) AAV serotype 9 vector system [8] with the aim of overcoming the limits of AAV for packaging the NG-ABE8e expression cassette and expressing NG-ABE8e as a full-length protein (Fig. 1b and Additional file 1: Fig. S2). The final constructs encoding *MAPT*-P301S-targeting or *Rosa26*-targeting sgRNAs are referred to as tsAAV-NG-ABE8e-*MAPT* and tsAAV-NG-ABE8e-*Rosa26*, respectively.

Eight weeks after intracranial injection (Additional file 1: Fig. S2b), we collected the hippocampi and evaluated the assembly of NG-ABE8e-N-terminus (NT) and –C-terminus (CT) sequences by measuring its expression level (Additional file 1: Fig. S2c). Next, we extracted hippocampal genomic DNA and measured the adenine base editing efficiency. NG-ABE8e-*MAPT* induced precise A-to-G base substitutions, converting 11-A (*MAPT*-P301S) to the WT sequence with a frequency of 5.7% ± 0.4%, as assessed by targeted deep sequencing (Fig. 1b). Bystander editing at 13-A, located 13 nt distal from the 5′-end of protospacer was observed with a frequency of 0.35% ± 0.04%. In addition, tsAAV-NG-ABE8e-*Rosa26* as a control induced precise A-to-G base substitutions with a frequency of 14.1% ± 3% (Additional file 1: Fig. S3a). Bystander indels were not observed in any of the treated samples (Additional file 1: Fig. S3b and c).

We next investigated whether NG-ABE8e exhibits off-target nuclease activity in the hippocampi of PS19 mice. To determine the genome-wide specificity of the *MAPT*-targeting NG-ABE8e nuclease, we first carried out targeted deep sequencing at potential off-target sites in the human genome, which differed from the *MAPT* on-target site by up to two nucleotides. Potential sites were identified using the Cas-OFFinder program. The regions containing on-target and off-target sites were amplified using the primer pairs listed in Table S2. We found no evidence of off-target effect in either *MAPT*-edited or *Rosa26*-edited hippocampi of PS19 mice (Fig. 1c, Additional file 1: Fig. S4 and Table S3). Taken together, these results show that the NG-ABE8e nuclease targeted *MAPT* or *Rosa26* in a highly specific manner*.*

Next, to determine the effects of tsAAV-NG-ABE8e-*MAPT* treatment on the pathological features of PS19 mice, we measured both total and phosphorylated tau (phospho-tau) protein levels in the soluble and insoluble fractions of protein lysates from hippocampal samples, utilizing antibodies described in Table S4. We found a significant reduction of insoluble tau, although there was no significant change in the soluble fraction, except a reduction in the level of soluble phospho-tau (Ser396) (Fig. 1d, Additional file 1: Fig. S5 and S6). In particular, both the total tau and the phospho-tau levels were decreased in the insoluble fraction, indicating a reduction in the quantity of insoluble tau proteins following treatment with tsAAV-NG-ABE8e-*MAPT*. Moreover, we found reductions in the AT8-positive areas in the hippocampi of mice treated with tsAAV-NG-ABE8e-*MAPT* (Fig. 1e and Additional file 1: Fig. S7). This reduction of AT8 staining was related to the expression of NG-ABE8e-*MAPT* (Fig. 1f). A previous report showed that expression of tau proteins containing the P301 mutation makes cells more vulnerable to be seeded with exogenous tau fibrils, which are present in insoluble fractions [9]. In line with this finding, we speculate that the NG-ABE8e-mediated correction of the P301S mutation might protect neurons from tau propagation and insoluble tau aggregation. In addition, neither the level of *MAPT* expression nor gliosis was affected by tsAAV-NG-ABE8e-*MAPT* treatment (Additional file 1: Fig. S8). These results suggest that correction of the targeted *MAPT*-P301S mutation by NG-ABE8e-*MAPT* alleviates insoluble tau aggregation in neurons.

Notably, tsAAV-NG-ABE8e-*MAPT* treatment improved the cognitive function of PS19 mice, as assessed by the Morris water maze test and the passive avoidance test (PAT) (Fig. 1g and h, Additional file 1: Fig. S9). These results demonstrate that such treatment improved the spatial learning memory and contextual memory of PS19 mice.

A limitation of our study is that we only targeted the hippocampus in PS19 mice. As some regions in the cortex also exhibit tauopathy in PS19 mice, there might be unidentified effects from pathological tau proteins in the untreated cortex. Widespread transduction of AAV into the whole brain via ventricular injection in P0 pups [10], or the use of PHP.eB AAV [11], could be alternative strategies for examining the therapeutic effects of NG-ABE in more depth in our future studies. Additionally, the gene editing frequency we reported might be underestimated within the neuronal population of interest. This may arise from the extraction of genomic DNA from the entire hippocampus, which includes a mixture of both neuronal and non-neuronal cells. Based on the recently reported cell atlas of the mouse brain, 48.12% of the cells in the hippocampus are neurons [12]. Given this information, we speculate that our actual neuronal gene editing efficiency could potentially achieve approximately 10%–11%. Further investigations are needed to determine the neuronal transduction efficiency of tsAAV in the hippocampus of PS19 mice to evaluate the base editing frequencies in neuronal cells and to confirm the replicability of our results.

In conclusion, we showed significant decreases in the level of insoluble tau proteins and staining of tau inclusions in neuronal cell bodies, without any change in *MAPT* expression or the total tau protein level. For clinical applications, it would be of benefit to reduce tau aggregation specifically, without down-regulation of the overall level of endogenous tau. Our results support that the NG-ABE8e-mediated targeted mutation correction could be a potential strategy for treating tauopathy-related neurodegenerative diseases. In addition, in vivo targeted adenine base editing via delivery of tsAAV-NG-ABE8e will broaden the range of therapeutic targets for various neurodegenerative disorders.

### Supplementary Information

**Additional file 1: Fig. S1.** Adenine base editing frequencies induced by NG-ABE8e. **Fig. S2.** Intracranial delivery of tsAAV-NG-ABE8e into the hippocampus of PS19 mice. **Fig. S3.** RNA trans-splicing AAV encoding NG-ABE8e for targeted adenine base editing. **Fig. S4.** Genome-wide specificity of NG-ABE8e. **Fig. S5.** Representative image of immunoblot using anti-tau antibody between different lysis fractions. **Fig. S6.** Tau protein levels in soluble fraction of hippocampus. **Fig. S7.** Representative images and quantification of phospho-tau (AT8) staining of the mouse hippocampus. **Fig. S8.** The level of *MAPT* gene expression and gliosis. **Fig. S9.** Results from the Probe test of Morris water maze. **Table S1.** The sgRNA target sequences in this study. **Table S2.** List of primers used for targeted deep sequencing. **Table S3.** Potential off-target sites of NG-ABE8e targeted to *MAPT* or *Rosa26* identified by Cas-OFFinder. **Table S4.** Information of antibodies used in this study. Materials and Methods.

## Data Availability

The deep sequencing data from this study have been submitted to the NCBI Sequence Read Archive under accession number PRJNA909014. The data that support the findings of this study are available from the corresponding author upon reasonable request.

## Abbreviations

*MAPT*: Microtubule-associated protein tau
tsAAV: Trans-splicing adeno-associated virus
hSyn-1: Human synapsin-1
