# Supplementary material for: CRISPR base editing-mediated correction of a tau mutation rescues cognitive decline in a mouse model of tauopathy
Source: Transl Neurodegener. 2024 Apr 12;13:21. doi: 10.1186/s40035-024-00415-y (PMC11010288; doi:10.1186/s40035-024-00415-y)
Supplement: Supplementary file 1 — Additional file 1: Fig. S1. Adenine base editing frequencies induced by NG-ABE8e. Fig. S2. Intracranial delivery of tsAAV-NG-ABE8e into the hippocampus of PS19 mice. Fig. S3. RNA trans-splicing AAV encoding NG-ABE8e for targeted adenine base editing. Fig. S4. Genome-wide specificity of NG-ABE8e. Fig. S5. Representative image of immunoblot using anti-tau antibody between different lysis fractions. Fig. S6. Tau protein levels in soluble fraction of hippocampus. Fig. S7. Representative images and quantification of phospho-tau (AT8) staining of the mouse hippocampus. Fig. S8. The level of MAPT gene expression and gliosis. Fig. S9. Results from the Probe test of Morris water maze. Table S1. The sgRNA target sequences in this study. Table S2. List of primers used for targeted deep sequencing. Table S3. Potential off-target sites of NG-ABE8e targeted to MAPT or Rosa26 identified by Cas-OFFinder. Table S4. Information of antibodies used in this study. Materials and Methods. [file 40035_2024_415_MOESM1_ESM.docx]

**CRISPR base editing-mediated correction of a tau mutation rescues cognitive decline in a mouse model of Tauopathy**

Min Sung Gee^1,#^, Eunji Kwon^2,#^, Myeong-Hoon Song^2^, Seung Ho Jeon^1^, Namkwon Kim^1^

 Jong Kil Lee^1*^, Taeyoung Koo^1,2,3*^

1 College of Pharmacy, Kyung Hee University, Seoul, 02447, Republic of Korea

2 Department of Biomedical and Pharmaceutical Sciences, Graduate School, Kyung Hee University, Seoul, 02447, Republic of Korea

3 Department of Pharmaceutical Sciences, College of Pharmacy, Kyung Hee University, Seoul, 02447, Republic of Korea

^#^ These authors contributed equally to this work.

*Correspondence to Jong Kil Lee ([jklee3984@khu.ac.kr](mailto:jklee3984@khu.ac.kr)) or Taeyoung Koo ([taeyoungkoo@khu.ac.kr](mailto:taeyoungkoo@khu.ac.kr)).

**
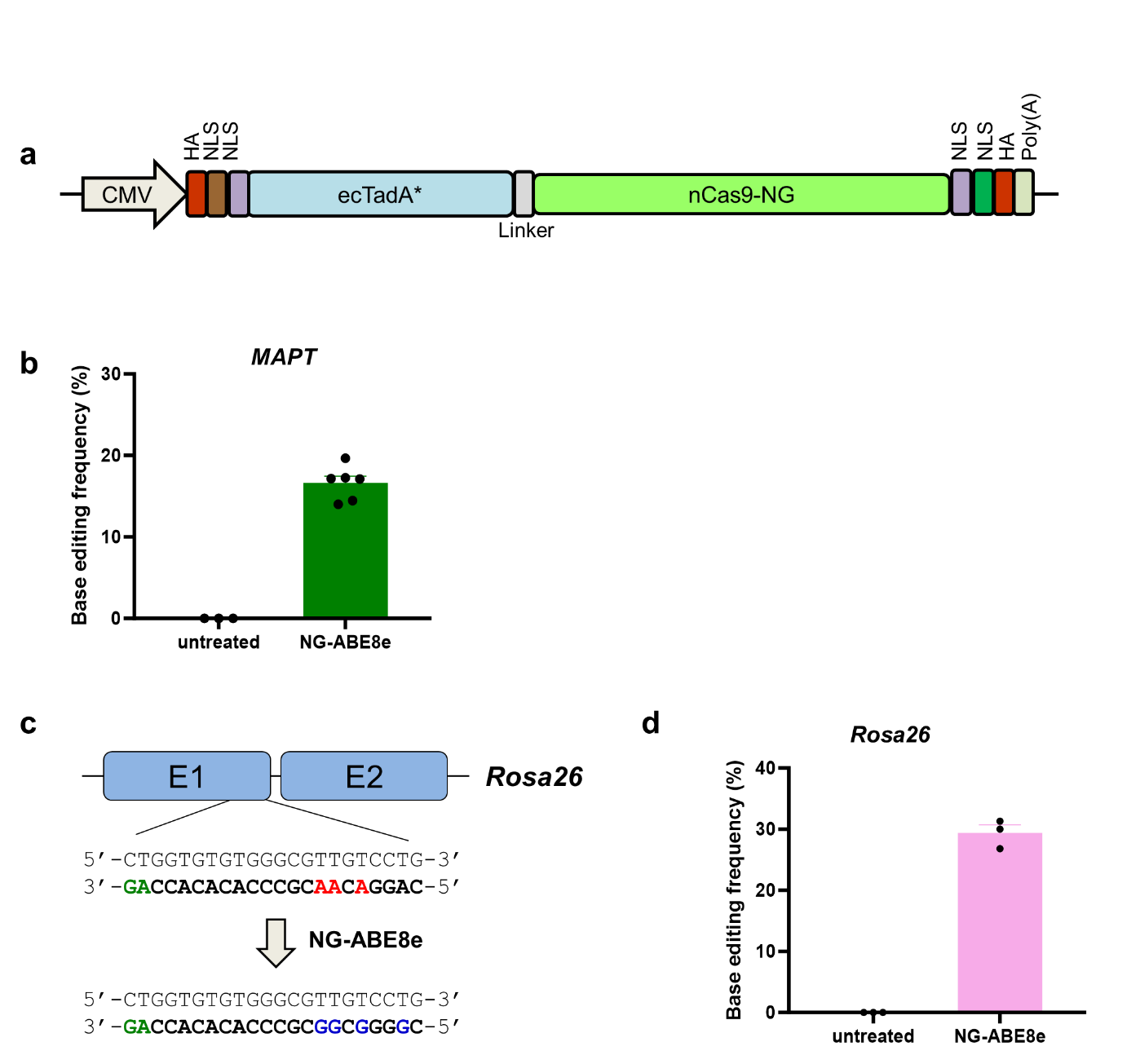
**

**Fig. S1. Adenine base editing frequencies induced by NG-ABE8e**. **a** Diagram of NG-ABE8e; NLSs are present at both the C- and N-termini. **b** adenine base editing frequencies at the *MAPT* target site in 293T*-*P301S cells. Error bars indicate s.e.m. (*n =* 6). **c** sgRNA was designed to target *Rosa26*. PAM sequences are shown in green, the targeted nucleotide in red, and base edited nucleotides in blue. **d** adenine base editing frequencies at the *Rosa26* target site in NIH3T3 cells. Error bars indicate s.e.m. (*n =* 3).

**
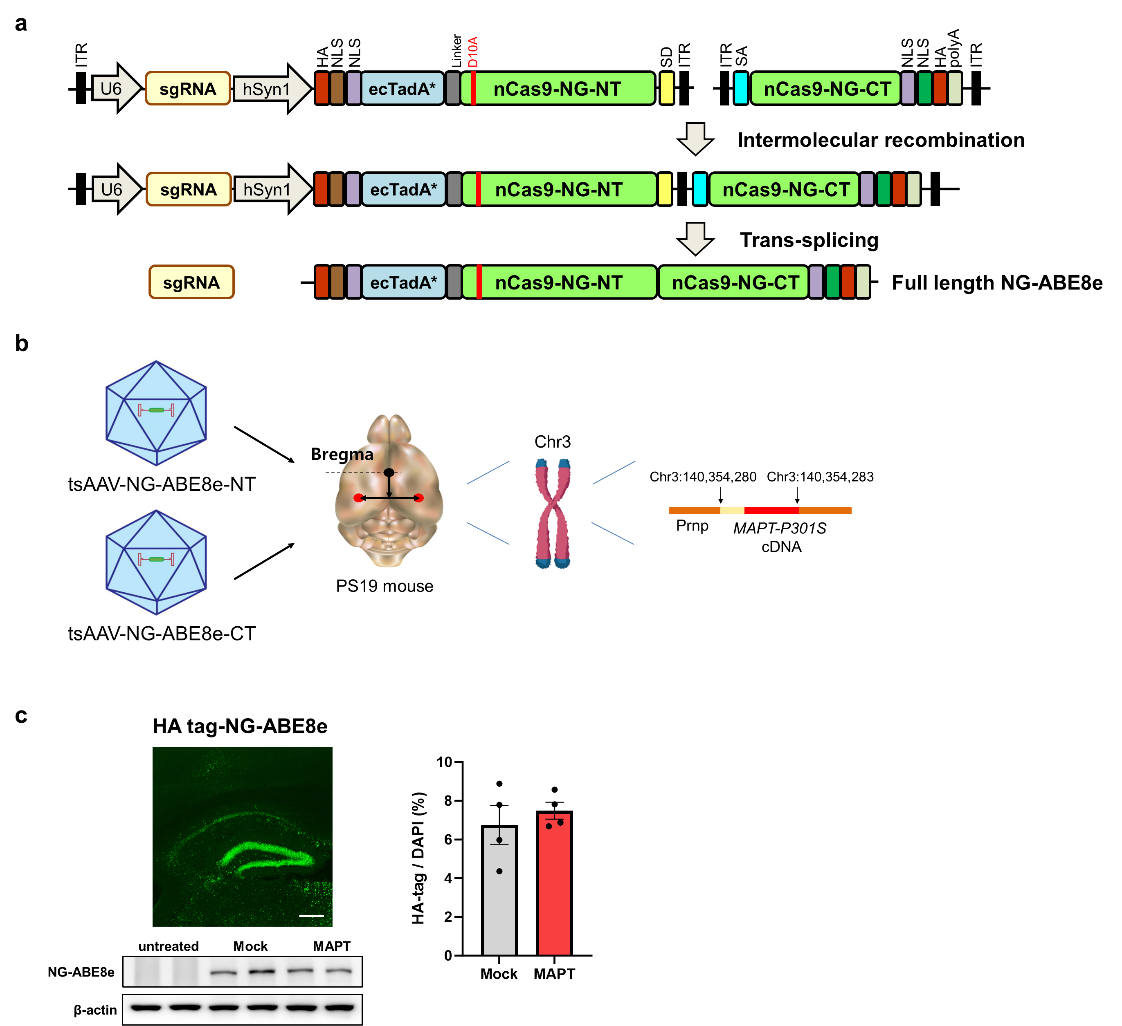
**

**Fig. S2. Intracranial delivery of tsAAV-NG-ABE8e into the hippocampus of PS19 mice. a** Schematic diagram of tsAAV-mediated assembly of NG-ABE8e following delivery into the hippocampi of PS19 mice. This system comprised two separate components: The tsAAV-NT includes the N-terminal segment of NG-ABE8e, deliberately omitting the polyA sequence and stop codon to prevent its expression. This design effectively eliminated the possibility of partial protein expression. The N-terminal NG-ABE8e sequences are conjugated with a synthetic splicing donor. In addition, this vector contains a sgRNA expression cassette. The tsAAV-CT encodes second half of NG-ABE8e conjugated with a synthetic splicing acceptor, followed by a poly A. We employed the hSyn-1 promoter [1] to specifically drive the expression of NG-ABE8e in the cortical neurons of PS19 mice. **b** Following intracranial injection of tsAAV-NG-ABE8e-NT and -CT vectors (1x10^10^ vg of each vector in 2 µl total), the NG-ABE8e targets the *MAPT-P301S* mutation or *Rosa26* in the PS19 mouse. **c** Representative images of hippocampus that has been stained with anti-HA-tag antibodies at eight weeks post-injection of tsAAV-NG-ABE8e-*MAPT*. HA-tag staining indicates expression of the NG-ABE8e fusion protein (scale bar, 300 µm). Immunoblotting shows the levels of HA-tagged NG-ABE8e and β-actin in the hippocampus. Relative HA-tag positive area (%) is also measured from immunohistochemistry. Error bars indicate SEM (*n* = 4). Mock; tsAAV-NG-ABE8e-*Rosa26* treated group, MAPT; tsAAV-NG-ABE8e-*MAPT* treated group.


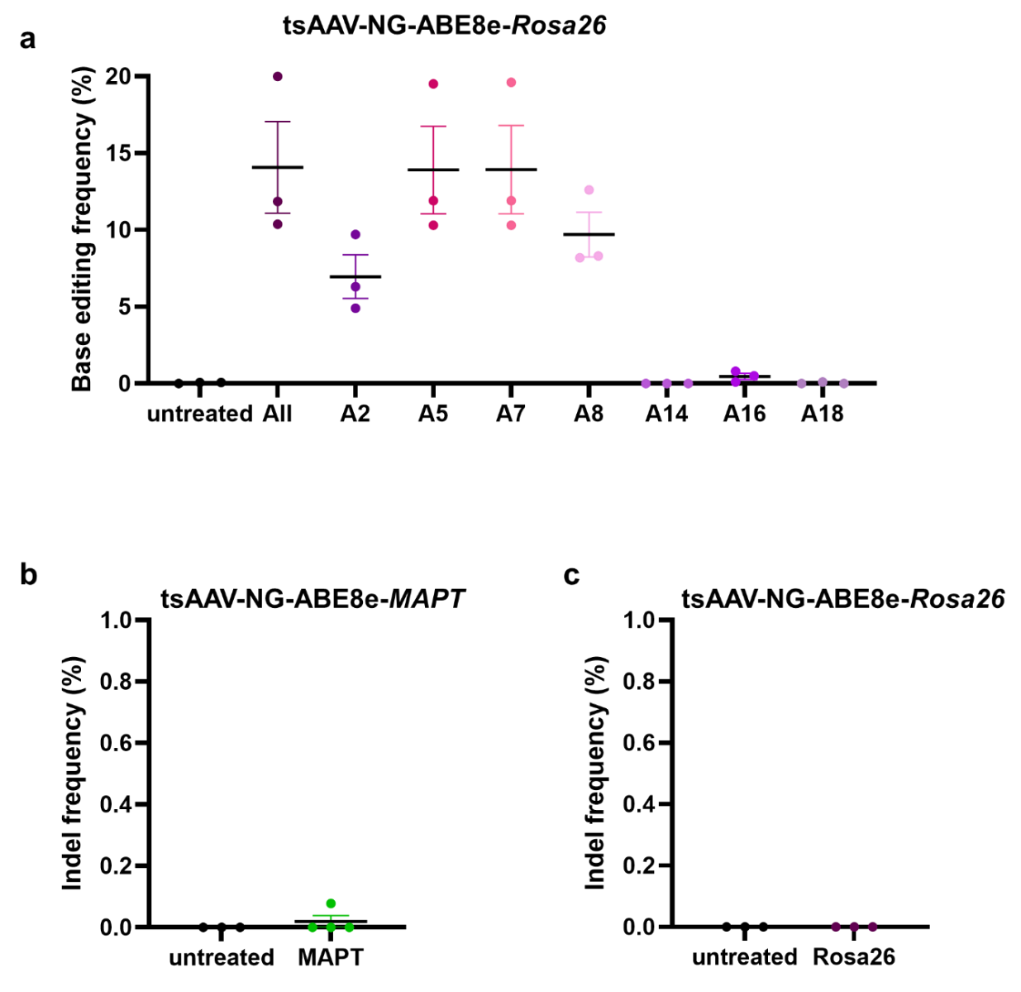


**Fig. S3. RNA trans-splicing AAV encoding NG-ABE8e for targeted adenine base editing. a** Base editing frequencies induced by tsAAV-NG-ABE8e-*Rosa26* in the hippocampus of PS19 mice at eight weeks after intracranial injection. **b and c** Indel frequencies induced by tsAAV-NG-ABE8e-*MAPT* **(b)** or -*Rosa26* **(c)** in the hippocampus of PS19 mice at eight weeks after intracranial injection. Error bars indicate s.e.m. (*n =* 3~4).


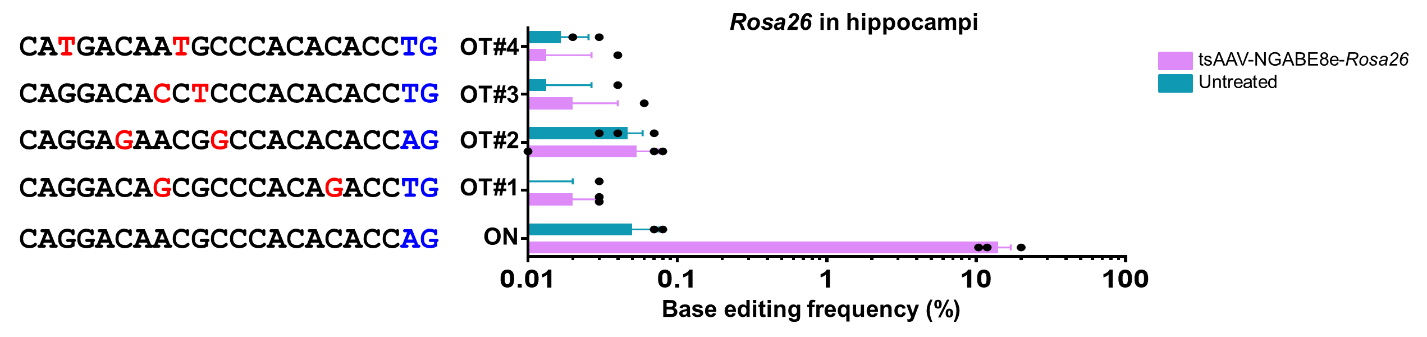


**Fig. S4. Genome-wide specificity of NG-ABE8e.** Genomic DNA isolated from the hippocampi of PS19 mice eight weeks after injection of tsAAV-NG-ABE8e-*Rosa26* was subjected to targeted deep sequencing. Mismatched nucleotides are shown in red, and PAM sequences in blue. ON, on-target site; OT, off-target site. Error bars indicate s.e.m. (*n =* 3).


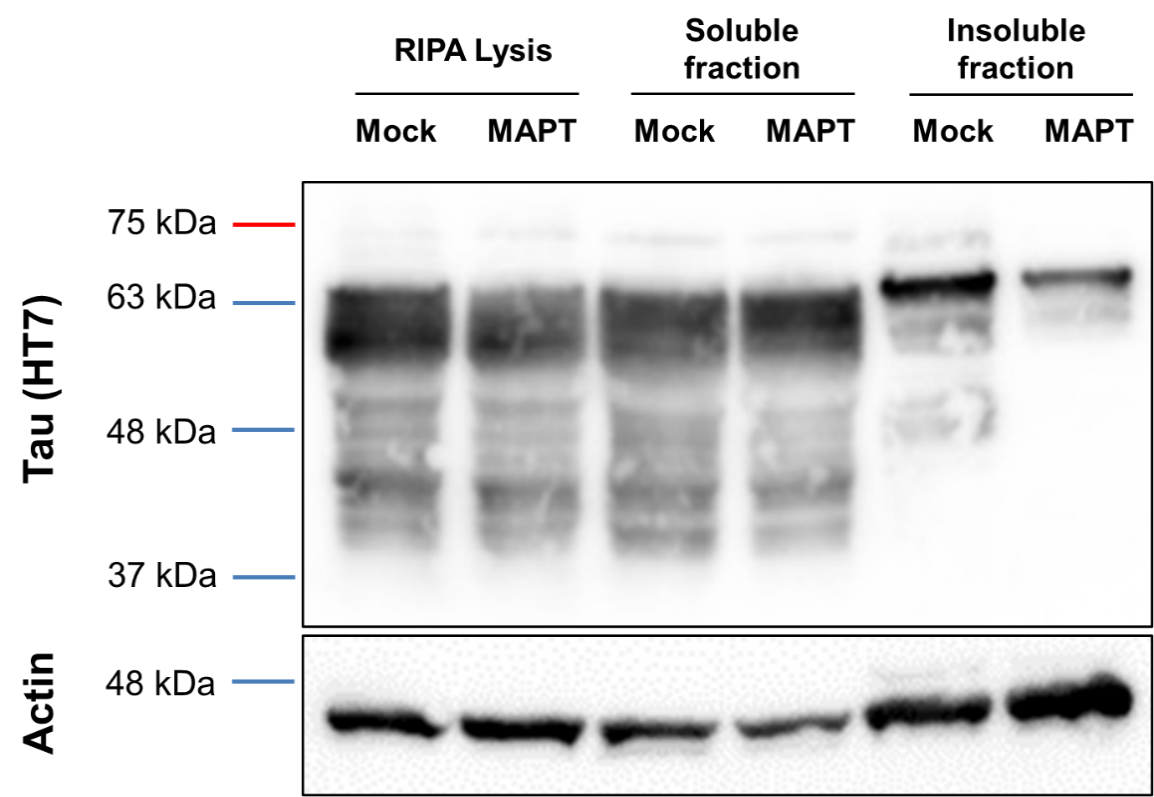


**Fig. S5. Representative image of immunoblot using anti-tau antibody between different lysis fractions.** PS19 mouse hippocampus samples were divided into NP-40 soluble and insoluble fraction. RIPA-lysed soluble protein sample was also prepared for comparison. Three fractions were immunoblotted using anti-tau antibody (HT7). The insoluble fraction samples were loaded with extra-high concentration to show the immunoblot patterns with other lysis fraction together in a same blot. Mock, tsAAV-NG-ABE8e-*Rosa26* treated group; MAPT, tsAAV-NG-ABE8e-*MAPT* treated group.


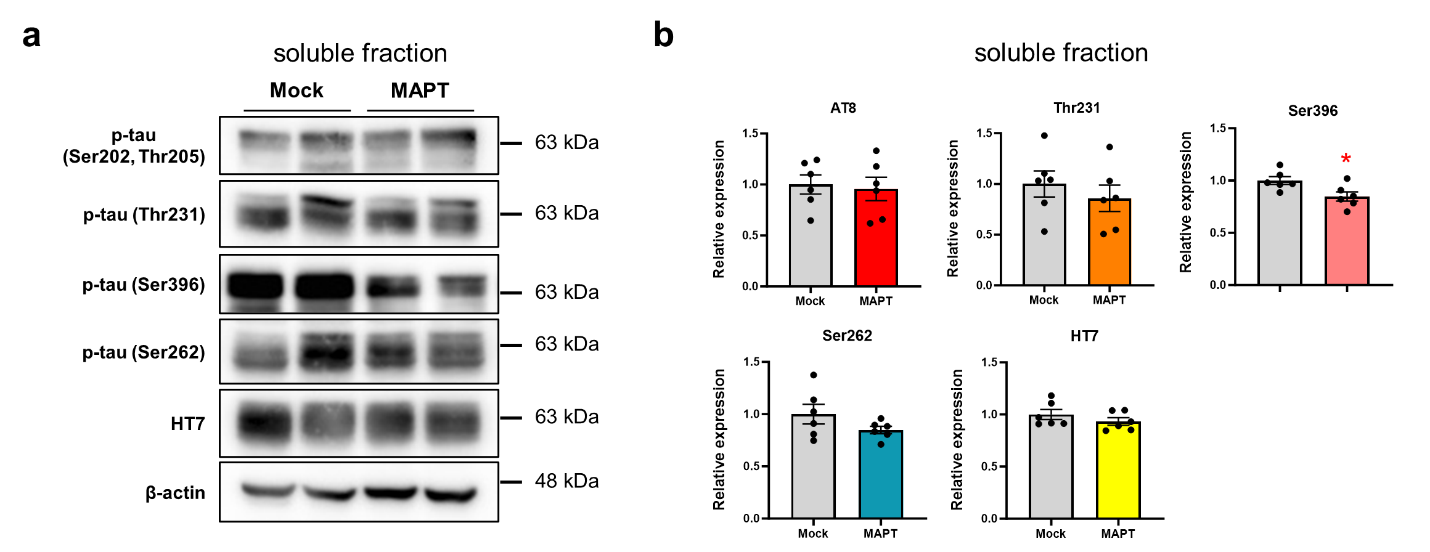


**Fig. S6. Tau protein levels in soluble fraction of hippocampus. a** Hippocampal protein from 9-month-old PS19 mice treated with tsAAV-NG-ABE8e-*MAPT* or -*Rosa26* was divided into two fractions (NP-40 soluble- and insoluble- fractions). tau protein levels were measured by immunoblotting using anti-phospho-tau antibodies (p-tau) and an antibody recognizing total tau (HT7). **b** Quantification of tau levels in soluble fractions (**P*<0.05 vs. mock control, Student′s *t*-test). Error bars indicate s.e.m. (*n =* 6).

**
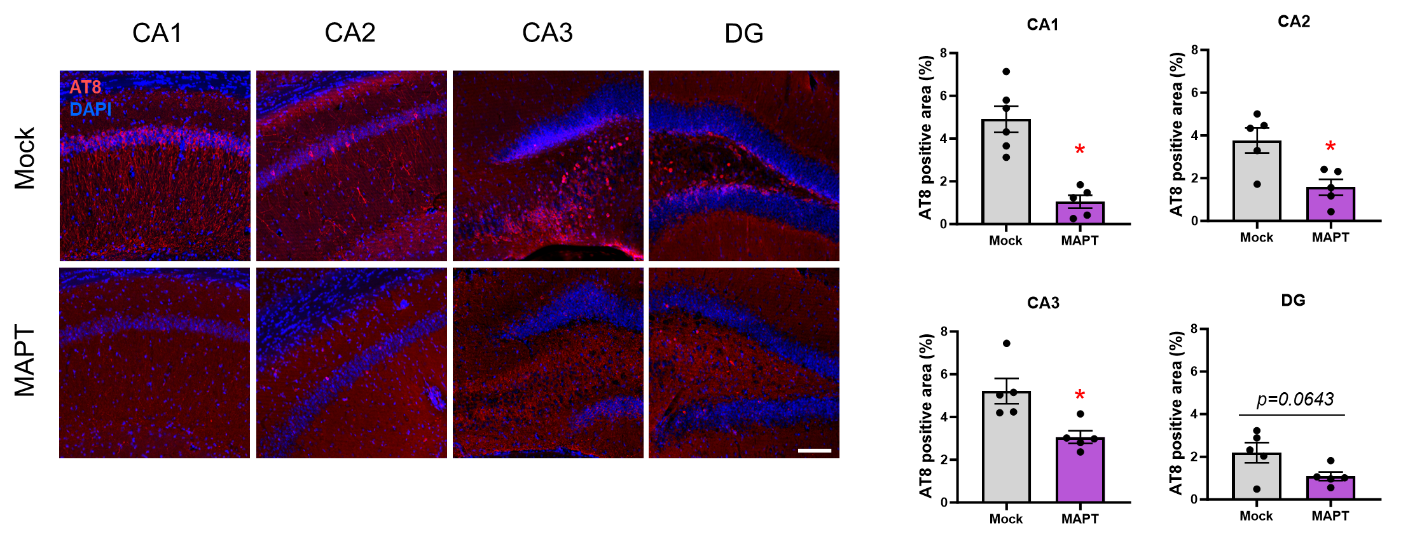
**

**Fig. S7. Representative images and quantification of phospho-tau (AT8) staining of the mouse hippocampus.** Mouse brains from both Mock and MAPT groups eight weeks after injection were stained with anti-phospho-tau (AT8) antibody (**P*<0.05 vs. mock control, Student′s *t-*test, scale bar, 100 µm). Error bars indicate s.e.m. (*n =* 5~6) Mock, tsAAV-NG-ABE8e-*Rosa26* treated group: MAPT, tsAAV-NG-ABE8e-*MAPT* treated group.

**
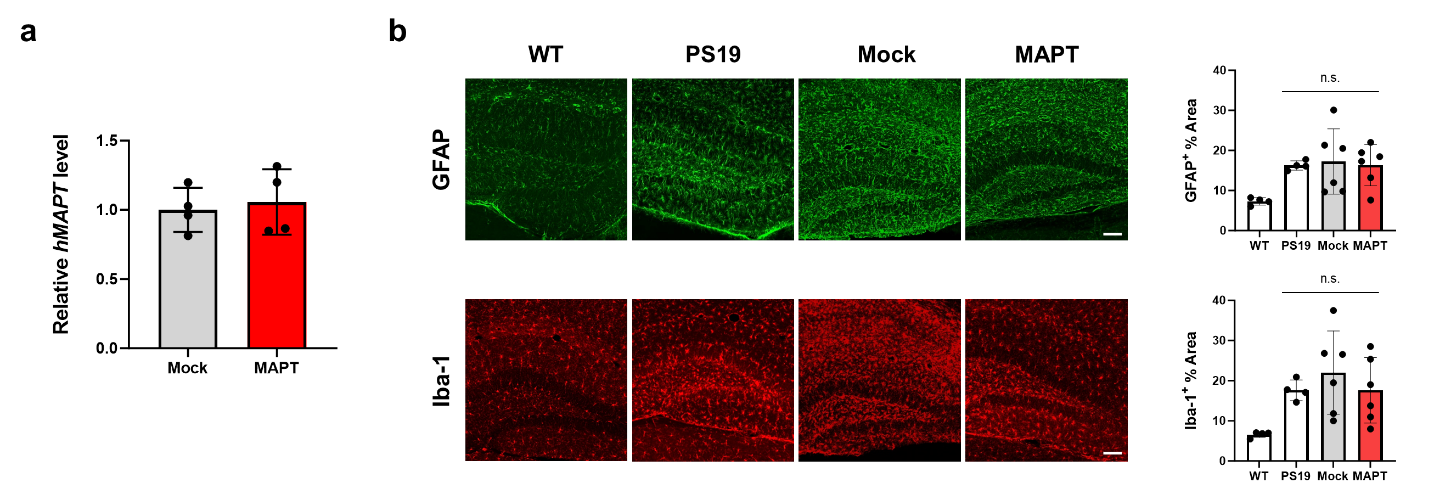
**

**Fig. S8. The level of *MAPT* gene expression and gliosis.** Eight weeks after intracranial injection of tsAAV-NG-ABE8e-*Rosa26* or -*MAPT* into the hippocampus of PS19 mice, brains were collected and analyzed. **a** The level of human *MAPT* gene expression was analyzed using qRT-PCR in Mock and MAPT group. Data are expressed as fold change with respect to the mean of the Mock group (*n* = 4). **b** Mouse brains were stained using anti-GFAP antibody (labeling astrocytes) and anti-Iba-1 antibody (labeling microglia). Hippocampus region was imaged using confocal microscopy; representative images are shown. Scale bar, 100 μm**.** Quantitative data are shown as mean ± SD (*n* = 4 for WT and PS19 groups; *n* = 6 for Mock and MAPT groups, One-way ANOVA). Mock, tsAAV-NG-ABE8e-*Rosa26* treated group; MAPT, tsAAV-NG-ABE8e-*MAPT* treated group. n.s; non-significant.


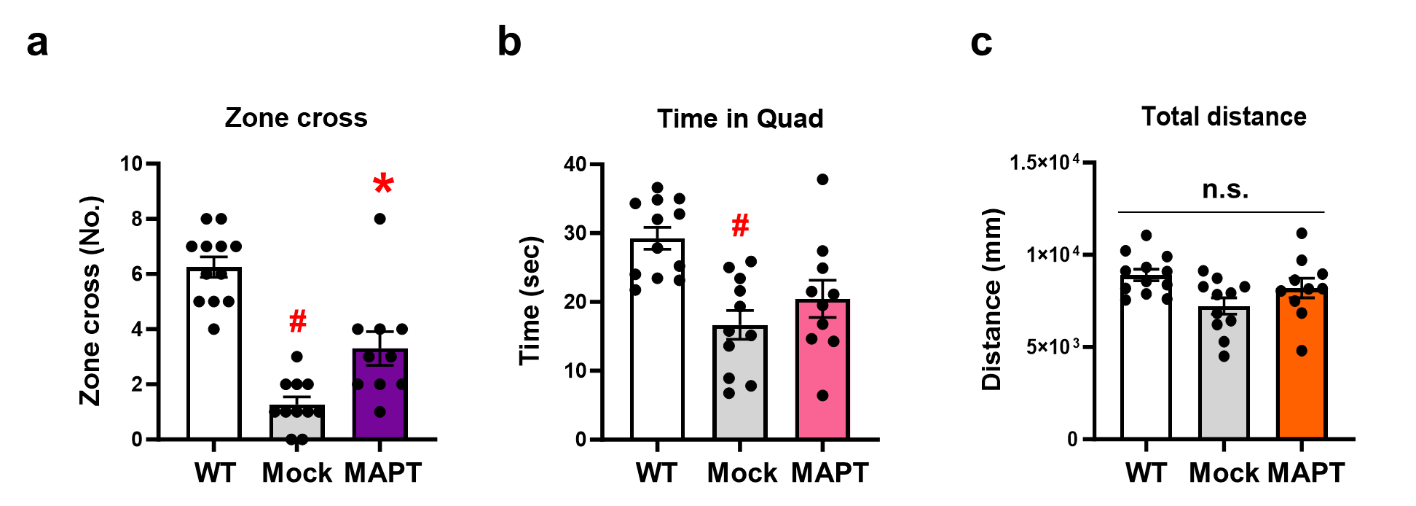


**Fig. S9.** **Results from the Probe test of Morris water maze.** Water maze test was performed eight weeks after intracranial injection of tsAAV-NG-ABE8e-*MAPT* or -*Rosa26* into the hippocampi of PS19 mice (WT *n* = 12; Mock *n* = 11; MPAT *n* = 10). After training period, **(a)** number of the target zone cross, **(b)** occupied time in the target quadrant, and **(c)** the total distance traveled were recorded during a 60-s probe test. Data are shown as mean ± s.e.m (#*P*<0.05 vs. WT; **P*<0.05 vs. mock control, One-way ANOVA, n.s; non-significant).

**Table S1.** The sgRNA target sequences in this study are shown in black and PAM sequences in blue.

| **Gene** | **Length of gRNA** | **Target sequence** | **Base editing (%)** |
| --- | --- | --- | --- |
| *MAPT* | 19mer | CCGCCTCCCGAGACGTGTT**TG** | 16.6±0.8 |
| **Gene** | **Length of gRNA** | **Target sequence** | **Base editing (%)** |
| *Rosa26* | 20mer | CAGGACAACGCCCACACACC**AG** | 29.4±1.3 |

**Table S2.** List of primers used for targeted deep sequencing.

|  | **1^st^ PCR** | | **2^nd^ PCR** | |
| --- | --- | --- | --- | --- |
| **ID** | **Forward (5′ to 3′)** | **Reverse (5′ to 3′)** | **Forward (5′ to 3′)** | **Reverse (5′ to 3′)** |
| *MAPT*-ON | CGCTGGAAATCACTCACACT | CATCTGCCCTATTCTGTCCAC | ACACTCTTTCCCTACACGACGCTCTTCCGATCTCGCATGTCACTCATCGAAAG | GTGACTGGAGTTCAGACGTGTGCTCTTCCGATCTGCACTCACACCACTTCCTAA |
| *MAPT*-OT1 | ATCCAGCTCTGAGAACCGGA | CCCCACTCTGCTTCCTTCG | ACACTCTTTCCCTACACGACGCTCTTCCGATCTCCCCTGAGGCTCCGTGTATT | GTGACTGGAGTTCAGACGTGTGCTCTTCCGATCTCTCTGCCTTCCAACTCTCGG |
| *MAPT*-OT2 | CTCTGAGTGGCCTTACCTTTAG | TGCTAGGCTCCTCTCTACAA | ACACTCTTTCCCTACACGACGCTCTTCCGATCTGGTATTTCCTCAGTGATAAGGC | GTGACTGGAGTTCAGACGTGTGCTCTTCCGATCTTGTAACCAGGGCATGCTTCT |
| *MAPT*-OT3 | AACGGGCCTCCTTCTCAGTA | TGTATCCTTCCGGTCCCCAA | ACACTCTTTCCCTACACGACGCTCTTCCGATCTCCTGTGGGGTTGAGAACGTC | GTGACTGGAGTTCAGACGTGTGCTCTTCCGATCTCCCTGTTCCCCAAAGCTCTC |
| *MAPT*-OT4 | GAGGAGAGACAACACACGGG | ATGGAGACTGAGGGAGCACA | ACACTCTTTCCCTACACGACGCTCTTCCGATCTGACACACCTGCCACTCCTG | GTGACTGGAGTTCAGACGTGTGCTCTTCCGATCTGCCCAGACGCCAAATGAAAG |
| *MAPT*-OT5 | GCAAGAGAAGGAGCTGCGA | GGGCTTGGAGGAATGGCTTC | ACACTCTTTCCCTACACGACGCTCTTCCGATCTCTCCTTCTGCCAGACTTGAGC | GTGACTGGAGTTCAGACGTGTGCTCTTCCGATCTACATGCTGGCAAAACACCCA |
| *MAPT*-OT6 | AACCCTCTCATGCATGCCAC | GACTCTTCCTGTTGCCTCCAG | ACACTCTTTCCCTACACGACGCTCTTCCGATCTGAGGTAATGGGCGCTCTGAG | GTGACTGGAGTTCAGACGTGTGCTCTTCCGATCTTGTGCAGGCTGTGTGACATT |
|  |  |  |  |  |
| *Rosa26*-ON | CCAAAGTCGCTCTGAGTTGT | GGAGCGGGAGAAATGGATATG | ACACTCTTTCCCTACACGACGCTCTTCCGATCTCGGGAGTCTTCTGGGCAGGCTTAA | GTGACTGGAGTTCAGACGTGTGCTCTTCCGATCTCCGAGGCGGATCACAAGCAA |
| *Rosa26*-OT1 | CCAGGGTAGATCCCCTGATT | TGCCCACCCACAGATACTTT | ACACTCTTTCCCTACACGACGCTCTTCCGATCTTGGCACCCTTTTGAGGTG | GTGACTGGAGTTCAGACGTGTGCTCTTCCGATCTGGTCACTGGCTTAGTCCCTTC |
| *Rosa26*-OT2 | CTGCACCACCATCAGTTGAG | CTAGCACAGCAGCCATGTGT | ACACTCTTTCCCTACACGACGCTCTTCCGATCTGTGCCATCATGCCTGGTAA | GTGACTGGAGTTCAGACGTGTGCTCTTCCGATCTCCTGCCAACTCTCTGTCCAT |
| *Rosa26*-OT3 | CAGGACAACCAGGGCTACAC | GCACCCCTTTGTTGTTGAGT | ACACTCTTTCCCTACACGACGCTCTTCCGATCTCAGCATTTGAAAGGCAGAGG | GTGACTGGAGTTCAGACGTGTGCTCTTCCGATCTCCTCGCTCATCTCTCCTGAC |
| *Rosa26*-OT4 | CCCCCACTAGGGGATTTTTA | GCCAAGGCAAGAACTTAGGG | ACACTCTTTCCCTACACGACGCTCTTCCGATCTCCTCCCTCCTACCTGTGACC | GTGACTGGAGTTCAGACGTGTGCTCTTCCGATCTCTTGCTGGTTTGCTCTCTGAC |

ON, On-target site; OT, off-target site.

**Table S3.** Potential off-target sites of NG-ABE8e targeted to *MAPT* or *Rosa26* identified by Cas-OFFinder.

| **Target** |  | **Location** | **Target sequence** | **Base editing frequency (%)** |
| --- | --- | --- | --- | --- |
| *MAPT* | ON | Chr17: 46010377 | CCGCCTCCCGAGACGTGT-T**TG** | 5.66±0.44 |
|  | OT1 | Chr7: 45333814 | CCGCCTCCCG**T**GACGTGT**CCCG** | 0.03±0.03 |
|  | OT2 | Chr6: 130834438 | CC**T**GCCTCCC**T**AGA**G**GTGTT**TG** | 0±0.0 |
|  | OT3 | Chr6: 120149658 | CC**T**CCTCCC**C**AGAC**T**GTGTT**TG** | 0±0.0 |
|  | OT4 | Chr11: 113198856 | CC**T**GCCTCC**A**GA**A**ACGTGTT**GG** | 0.05±0.03 |
|  | OT5 | Chr11: 32417860 | CCGCC**C**CCCGAGA**CA**GTGTT**TG** | 0±0.0 |
|  | OT6 | Chr10: 76275038 | CCG**C**CCTCCC**C**AGACGTG**C**T**CG** | 0±0.0 |

| **Target** |  | **Location** | **Target sequence** | **Base editing frequency (%)** |
| --- | --- | --- | --- | --- |
| *Rosa26* | ON | Chr6: 113075969 | CAGGACAACGCCCACACACC**AG** | 14.1±3.0 |
|  | OT1 | Chr12: 41847051 | CAGGACA**G**CGCCCACA**G**ACC**TG** | 0.02 ±0.0 |
|  | OT2 | Chr17: 56282870 | CAGGA**G**AACG**G**CCACACACC**AG** | 0.05 ±0.0 |
|  | OT3 | Chr9: 31095503 | CAGGACA**C**C**T**CCCACACACC**TG** | 0.02 ±0.0 |
|  | OT4 | Chr10: 91398646 | CA**T**GACAA**T**GCCCACACACC**TG** | 0.01 ±0.0 |

Mismatched nucleotides are shown in red, PAM sequences in blue, and DNA bulge in green. ON, on-target site; OT, off-target site.

**Table S4.** Information of antibodies used in this study.

| Target | Host | Source | Catalog No. | RRID | Application |
| --- | --- | --- | --- | --- | --- |
| HA-tag | Rabbit | Cell Signaling Technology | 3724 | AB_1549585 | WB/1:1000  IHC/1:500 |
| Tau (HT7) | Mouse | Invitrogen | MN1000 | AB_2314654 | WB/1:1000 |
| Phospho-tau (AT8; Ser202, Thr205) | Mouse | Invitrogen | MN1020 | AB_223647 | WB/1:1000  IHC/1:500 |
| Phospho-tau (Thr231) | Mouse | Invitrogen | MN1040 | AB_223649 | WB/1:1000 |
| Phospho-tau (Ser262) | Rabbit | Invitrogen | 44-750G | AB_2533743 | WB/1:1000 |
| Phospho-tau (Ser396) | Rabbit | Invitrogen | 44-752G | AB_2533745 | WB/1:1000 |
| β-actin (HRP) | Mouse | Santa Cruz Biotechnology | sc-47778 HRP | AB_2714189 | WB/1:5000 |
| Iba-1 | Rabbit | Wako | 019-19741 | AB_839504 | IHC/1:500 |
| GFAP | Chicken | Abcam | AB4674 | AB_304558 | IHC/1:500 |
| Rabbit IgG (HRP) | Mouse | Santa Cruz Biotechnology | sc-2357 | AB_628497 | WB/1:5000 |
| Mouse IgG (HRP) | Mouse | Santa Cruz Biotechnology | sc-516102 | AB_2687626 | WB/1:5000 |
| Mouse IgG (594) | Goat | Invitrogen | A-11032 | AB_2534091 | IHC/1:1000 |
| Rabbit IgG (488) | Goat | Invitrogen | A-11008 | AB_143165 | IHC/1:1000 |
| Chicken IgY (488) | Goat | Invitrogen | A-11039 | AB_2534096 | IHC/1:1000 |

*WB* western blot, *IHC* immunohistochemistry

**Materials and Methods**

**Construction of plasmids encoding NG-ABE8e and sgRNA**

We codon-optimized the sequences encoding NG-ABE8e (Addgene #138491); it was conjugated with two nuclear localization signal (NLS) sequences so that there would be two NLS sequences at both the N- and C-termini. For construction of AAV-NG-ABE8e vector plasmids, the sequences encoding evolved *E.coli* TadA (TadA*), the N-terminal half of Cas9 nickase (nCas9)-NG (2,142 bp, 714 bp amino acids) conjugated with a NLS sequence and HA tag, and the hSyn-1 promoter were cloned into an AAV ITR-based vector plasmid (pAAV-NG-ABE8e-NT). The sgRNA sequences targeting *MAPT* or *Rosa26* were also inserted in the pAAV-NG-ABE8e-NT vector and transcribed under the control of the U6 promoter. The sequence encoding the C-terminal half of nCas9 (1,959 bp, 653 amino acids) conjugated with a NLS sequence, an HA tag sequence, and the simian virus 40 poly A signal were cloned into an AAV ITR-based vector plasmid (pAAV-NG-ABE8e-CT). The sgRNA-encoding sequences are subcloned into the pU6-sgRNA vector plasmid, digested with BsaI (New England Biolabs). Sequences of the sgRNAs targeting the *MAPT* and *Rosa26* genes are listed in **Tables S1**.

**Cell culture and transfection**

HEK293T (ATCC, CRL-3216) and NIH3T3 (ATCC, CRL-1658) cells were maintained in Dulbecco′s Modified Eagle′s Medium (DMEM, Welgene, cat. no. LM001-05) supplemented with 100 units/ml penicillin (Welgene, cat. no. LS202-2), 100 µg/ml streptomycin, and 10% heat-inactivated fetal bovine serum (FBS, Welgene, cat. no. S 101-01). Cells were seeded into 24-well plates one day prior to transfection and transfected with the sgRNA plasmid (1500 ng) and the NG-ABE8e plasmid (500 ng) using 4 µl of PEImax (Polysciences, cat. no. 24765-2) or Jetprime (Polyplus, cat. no. 114-15). To evaluate prime editing efficiency, HEK293T-P301S cells were seeded into 24-well plates one day prior to transfection and transfected with the pegRNA plasmid (1500 ng) and the PE2 plasmid (500 ng) using 4 µl of PEImax (Polysciences, cat. no. 24765-2).

**Production and titration of AAV vectors**

To produce AAV vectors, they were pseudotyped in AAV9 capsids for transfection into cells or the hippocampi of PS19 mice. HEK293T cells were transfected with pAAV-NG-ABE8e-NT or pAAV-NG-ABE8e-CT, pAAVED2/9 encoding AAV2rep and AAV9cap, and helper plasmid. HEK293T cells were cultured in DMEM with 2% FBS. Recombinant pseudotyped AAV vector stocks were generated using polyethylenimine (PEI) coprecipitation with PEImax (Polysciences, cat. no. 24765-2) and triple-transfection with plasmids at a molar ratio of 1:1:1 in HEK293T cells. After 72 h of incubation, cells were lysed and particles were purified by iodixanol (Sigma-Aldrich) step-gradient ultracentrifugation. The number of vector genomes was determined by quantitative PCR.

**Targeted deep sequencing**

Genomic DNA was isolated from cells or whole hippocampi of PS19 mice using a DNeasy Blood & Tissue kit (Qiagen) according to the manufacturer’s instructions. On-target or off-target loci were amplified from 20~50 ng of genomic DNA for targeted deep sequencing. Deep-sequencing libraries were generated by PCR. The region of interest was amplified using the primer pairs listed in **Table S2.** For the targeted deep sequencing analysis of the *MAPT* gene in PS19 mice, we used human-specific *MAPT* primers to preclude amplification of the mouse *Mapt* gene. Additionally, the target site in the *MAPT* gene is distinguishable from the corresponding mouse sequence by three base pair mismatches. TruSeq HT Dual Index primers were used to label each sample. The PCR products were purified using Expin PCR SV (Geneall Biotechnology, Korea). Pooled libraries were subjected to paired-end sequencing using MiniSeq (Illumina).

**Mutation analysis**

Substitutions and indel frequencies were calculated following the procedure described in our previous study [2]. Briefly, A-to-G conversion ratios in the mutant sequences were determined to calculate base editing frequency. Wild-type and mutant sequences were discriminated based on the presence of the single nucleotide missense mutation in the allele. Sequences carrying substitutions or indels were counted as genome edited sequences. Genome editing frequencies are given in **Tables S1 and S3**.

**Animals**

PS19 mice carrying the P301S tau mutation and WT mice with the same background were purchased from Jackson Laboratory (Stock No: 008169). The mice were bred and maintained in individual ventilated cages with 12-h light/dark cycles. PS19 mice, which carry the human *MAPT* cDNA sequence with a P301S mutation. The transgene, under the control of mouse *Prnp* promoter, is inserted at chromosome 3 of mouse genome, resulting that the mice exhibit tau tangles and behavior impairments in aging [3-5].

**Stererotaxic surgery**

Prior to surgery, which was performed using stereotaxic instruments, mice were anesthetized by injection of a Ketamine-Rompun mixture (5 µl/g). Seven-month-old PS19 mice were divided into two groups, which respectively received hippocampal injections of tsAAV-NG-ABE8e-*MAPT* or -*Rosa26*. The mice received AAV injections into both sides of the hippocampus (anteroposterior, -2; mediolateral, ±2.2; and dorsoventral, -2 related to Bregma). In preparation for injection, tsAAV-NG-ABE8e-NT and tsAAV-NG-ABE8e-CT were mixed at a 1:1 ratio. The compounds were injected in a total volume of 2 µl, at an infusion rate of 0.4 µl/min into each side of the hippocampus. Each injection contained 1x10^10^ vg of each vector. Mice were sacrificed at 9-month-old. The experiment was approved by the Kyung Hee University Institutional Animal Care and Use Committee (IACUC, KHUASP-20-231)

**Behavior tests**

Eight weeks after the viral vector injection, 9-month-old mice were subjected to behavior tests. MWM tests were conducted following the procedure described in our previous study [6]. For the PAT, mice were dark-adapted for 30 min and then placed in the bright compartment of a PAT chamber. On day one, the latency times before the mice entered the dark compartment were then recorded, and the mice received a mild foot shock in the dark compartment. On day two, the same procedure was repeated, without a foot shock. Each behavior test was recorded. Two independent sets of behavioral experiment were conducted. All female and male mice were used in behavior tests, and the PS19 mice showing severe hunched back and limb paralysis, were excluded in behavior tests.

**Protein sample preparation**

To separate the NP-40 soluble/insoluble fractions, NP-40 lysis buffer-1 (10 mM Tris-HCl, 150 mM NaCl, 5 mM EDTA, 0.5% NP-40) containing the protease/phosphatase inhibitor cocktail was used. After homogenization and centrifugation, the supernatants were collected (NP-40 soluble samples). Next, the pellets were washed with NP-40 lysis buffer-1 and reconstituted with NP-40 lysis buffer-2 (NP-40 lysis buffer-1 containing 1% SDS and 0.5% sodium deoxycholate). After sonication and incubation on ice for 1 h, the lysates were centrifuged at 13,000 x g for 30-min and the supernatants were collected (NP-40 insoluble samples).

**Immunoblotting**

Immunoblotting was performed following the procedure described in our previous study [6]. Briefly, after SDS-polyacrylamide gel electrophoresis and transfer to PVDF membranes, the membranes were blocked with 5% skim milk and probed with primary antibodies at 4℃ overnight. The next day, the membranes were probed with corresponding secondary antibodies conjugated with horseradish peroxidase. The information of antibodies used in this study is described in **Table S4**.

**Immunohistochemistry and image analysis**

Mouse brains were collected after perfusion with phosphate buffered saline and fixed by incubation in a 4% paraformaldehyde solution. The brains were sliced at 30 µm thickness using a vibratome (VT1200S, Leica). Immunohistochemistry experiments were conducted following the procedure described in our previous study [6]. Stained brain slices were imaged using confocal microscopy (K1-Fluo, Nanoscope Systems). Z-stacked images were acquired at 1.5 µm intervals (for a total of 15 optical slices). Four hippocampal regions (CA1, CA2, CA3, and dentate gyrus (DG)) were imaged from one brain slice, and four brain slices were used to quantify results from each mouse. The information of antibodies used in this study is described in **Table S4**.

**Quantitative real-time polymerase chain reaction**

Total RNA was extracted from the hippocampus of mice with a Hybrid-R total RNA purification kit (GeneAll®, 305-101) in accordance with the manufacturer’s instructions. The NanoDrop™-2000c (ThermoFisher Scientific) was used to measure the concentration and purity of the RNA samples. cDNA was synthesized using TOPscript RT DryMIX (Enzynomics, RT200), according to the manufacturer’s instructions. cDNA samples were subjected to qRT-PCR using SYBR Green Mix (Enzynomics, RT500) and a CFX Connect real-time PCR system (Bio-Rad). The level of human *MAPT* gene expression was analyzed using qRT-PCR in Mock and MAPT group. Primers for qRT-PCR analysis of human *MAPT* gene expression were as follows: TTAGCAACGTCCAGTCCAAG (*hMAPT*-Forward), TCAGGTCAACTGGTTTGTAGAC (*hMAPT*-Reverse), TGAATACGGCTACAGCAACA (*GAPDH*-Forward), AGGCCCCTCCTGTTATTATG (*GAPDH*-Reverse). The PCR protocol was 95°C for 10 min; 95°C for 10 sec, 60°C for 10 sec, 72°C for 30 sec (40 cycles); 95°C for 10 sec. The measured expression level was normalized to the GAPDH level using the 2^-ΔΔCT^ method.

**Statistical analysis**

No statistical methods were used to predetermine sample size for *in vitro* or *in vivo* experiments. All group results are expressed as mean ± s.e.m., if not stated otherwise. Comparisons between groups were made using the two-tailed Student′s *t*-test or one-way ANOVA and Tukey post-hoc tests for multiple groups. Statistical significance as compared to untreated controls is denoted with * (*P* < 0.05), ** (*P* < 0.01), *** (*P* < 0.001) in the figures and figure legends. Statistical analysis was performed in Graph Pad PRISM 8.

**References**

1. Kügler, S., et al., *Neuron-specific expression of therapeutic proteins: evaluation of different cellular promoters in recombinant adenoviral vectors.* Mol Cell Neurosci, 2001. **17**(1): p. 78-96.

2. Choi, E., et al., *Expanded targeting scope of LbCas12a variants allows editing of multiple oncogenic mutations.* Mol Ther Nucleic Acids, 2022. **30**: p. 131-142.

3. Allen, B., et al., *Abundant tau filaments and nonapoptotic neurodegeneration in transgenic mice expressing human P301S tau protein.* J Neurosci, 2002. **22**(21): p. 9340-51.

4. Takeuchi, H., et al., *P301S mutant human tau transgenic mice manifest early symptoms of human tauopathies with dementia and altered sensorimotor gating.* PLoS One, 2011. **6**(6): p. e21050.

5. Sun, Y., et al., *The behavioural and neuropathologic sexual dimorphism and absence of MIP-3alpha in tau P301S mouse model of Alzheimer's disease.* J Neuroinflammation, 2020. **17**(1): p. 72.

6. Gee, M.S., et al., *A selective p38alpha/beta MAPK inhibitor alleviates neuropathology and cognitive impairment, and modulates microglia function in 5XFAD mouse.* Alzheimers Res Ther, 2020. **12**(1): p. 45.
